# Study on attractors during organism evolution

**DOI:** 10.1038/s41598-021-89001-0

**Published:** 2021-05-05

**Authors:** Andrzej Kasperski, Renata Kasperska

**Affiliations:** 1 Institute of Biological Sciences, Department of Biotechnology, University of Zielona Gora, ul. Szafrana 1, 65-516 Zielona Gora, Poland; 2 Faculty of Mechanical Engineering, University of Zielona Gora, ul. Szafrana 4, 65-516 Zielona Gora, Poland

**Keywords:** Cancer models, Evolutionary theory, Bioinformatics, Cancer models, Cancer models, Evolution, Software

## Abstract

The important question that arises during determining the evolution of organisms is whether evolution should be treated as a continuous process or whether groups of organisms fall into 'local' attractors during evolution. A similar question arises during considering the development of cells after cancer transformation. Answers to these questions can provide a better understanding of how normal and transformed organisms evolve. So far, no satisfactory answers have been found to these questions. To find the answers and demonstrate that organisms during evolution get trapped in 'local' attractors, an artificial neural network supported by a semihomologous approach and unified cell bioenergetics concept have been used in this work. A new universal model of cancer transformation and cancer development has been established and presented to highlight the differences between the development of transformed cells and normal organisms. An unequivocal explanation of cancer initialization and development has not been discovered so far, thus the proposed model should shed new light on the evolution of transformed cells.

## Introduction

Random genetic mutations and natural selection are the factors that, according to existing theories, act as driving forces of organism evolution^1,2^. These phenomena may point at the continuous or discontinuous character of evolution. When considering evolution as a discontinuous process, large differences between genomes of evolving groups of organisms should be visible. In this case, it can be said that whole groups of organisms should be trapped in different attractors and evolve inside them. The term 'attractor' means a configuration (a set of values for the variables) towards which the system evolves over time. After attaining an attractor a given configuration of a system is sufficiently stable to return to the original state after disappearing an eventual perturbation^3^. In this work, the attractors which trap genomes of organisms are termed 'genome attractors'. For all cells of trapped (in the genome attractor) organism, special gene expression programs (termed 'cell-fates') are activated that enlive and keep alive the whole organism.

Most animal studies have used single mitochondrial DNA genes to evaluate population or low-level taxonomic relationships^4–7^. One of the most useful genes for phylogenetic reconstruction is cytochrome b, that is commonly used in systematic research to address many taxonomic level divergences^8,9^. Cytochrome b, alone or supported by other data sets (for example nuclear ribosomal rRNA gene, cytochrome oxidase I gene (COI), complete mitochondrial DNA), can yield phylogenetic trees that are in agreement with well-established phylogeny^9–16^. Research indicates that the use of cytochrome b is superior to COI when one locus is to be used as a standard for mammalian species phylogeny and identification^16^. Cytochrome b is very useful as a 'fingerprint' of organisms because it can harbour very few mutations due to the stringent structural and physiological links it obeys. This implies all the observed mutations are 'function preserving' and thus 'fitness independent', so that they can be considered as evolutionary neutral mutations only working as 'time keeping' measuring the evolution timing between two species. For these reasons, cytochrome b sequences of the selected groups of organisms have been examined in this work to check trapping the evolving groups of organisms in genome attractors. Especially important for checking genome attractors is the ability to use cytochrome b sequence variability in comparison of organisms in the same genus or the same family^17,18^. An example of using cytochrome b alone as a molecular marker can be establishing phylogenetic relationship at various levels within the fish family *Cichlidae*^9^. The obtained trees (as a result of analysis based on cytochrome b alone) have been consistent with the trees obtained as a result of the extended (total) analysis^9^. Other authors have presented that a partial DNA sequence of cytochrome b can be sufficient for animal identification. This has been demonstrated on the example of the identification of the remains of endangered animals and species endemic to Taiwan (i.e. clouded leopards, leopard cats, lions, tigers, water buffalos and selected Formosans)^19^.

The reconstruction of evolution on genetic levels usually requires applying a distance correction to take into account the impact of an intermediate/invisible stages of evolution. For this reason, the selection of a stochastic model (i.e. correction model) for estimating real evolutionary distance is required. These correction models include for example the Poisson, Dayhoff, Jones-Taylor-Thornton (JTT) models that can be used, inter alia, in Neighbor Joining and Maximum Likelihood methods^20^. Before calculation of distances between sequences, the sequences have to be aligned. To align sequences the appropriate method should be selected and parameterized. In this way, various results can be obtained and different conclusions regarding evolution can be drawn depending on the used methods and their parameterization. As a result, it can be said that a cloud of uncertainty covers over the real truth about evolution.

During organism evolution, macro-cellular evolution and micro-cellular evolution can be distinguished. Macro-cellular evolution is driven by genome re-organization, while micro-cellular evolution is driven by gene mutation and/or epigenetic function^21,22^. In this article the continuous/discontinuous character of evolution (with the consequent existence of macro-evolution processes separated from micro-evolution) has been investigated by means of a neural network inspired approach. The new idea presented in this work is to code the individual amino-acids of the cytochrome b sequences of organisms in many different ways. The aim of the different coding individual amino-acids was to put into account and neutralize the influence of different manners of amino-acid coding on the recognized by neural network evolutionary similarities. Additionally, the semihomologous approach has been used to validate the results. Studies of evolution using the semihomologous approach offer new possibilities related to take into account the similarities at the codon level^23–27^.

In this article, the evolution of normal organisms has been also compared with transformed cell development. New evolutionary models are proposed to reconcile macro-cellular evolution and micro-cellular evolution in view of cancer development. In one of the newest exemplary models, all individual molecular mechanisms are classified as genomic/environmental interactions that lead to macro-cellular evolution, followed by micro-cellular evolution to grow the cancer cell population^28,29^.

The article is organized as follows: firstly the methods and theoretical bases are listed, including a description of the neural network implementation and semihomologous approach. Secondly, selected aspects of evolution of normal organisms and transformed cells are given attention to. Lastly, the research conclusions are presented.

## Materials and methods

Cytochrome b amino-acid sequences selected for this study were taken from the protein databases NCBI and Protein BLAST.

### Design and teaching the artificial neural network

The artificial neural network (ANN) has been designed as a full synapse three layer neural network and it has been taught in a similar way as it is presented in^18^. These three layers (i.e. the input, hidden and output layer) are composed of neurons, all having the same characteristics. Each layer has been implemented as a sigmoid layer and transfers the input pattern to the output pattern by executing a sigmoid transfer function (y = 1/(1 + exp(-x))) that gives a smooth output limitation within the range 0 and 1^30^. The layers are connected using synapses that permit a pattern to be passed from the input layer to the hidden layer and next from the hidden layer to the output layer. In the implemented neural network, all the nodes of the input layer are connected with all the nodes of the hidden layer and all the nodes of the hidden layer are connected with all the nodes of the output layer. Because the number of amino-acids (AA) in the cytochrome b sequences is usually not bigger than 400 for almost all organisms, it has been assumed that the length of sequences in the input of the neural network is equal to 400 AA. In order to obtain a length equal to 400, the sequences have been aligned by addition of the "−" characters at the end of sequences or have been cut. After the alignment, each sequence has been converted to the binary form by changing each character to a random generated five-positional binary number. After converting, the new binary form of sequence (which is entered into the neural network input layer) has a length of 2000 and the number of neurons in the neural network input layer is equal to 2000 (i.e. n = 2000). The number of neurons in the output layer (k) is equal to the number of organisms used for ANN teaching (i.e. k = 36). The number of neurons in the hidden layer (m) is calculated by the geometric pyramid rule^31^, i.e. m = sqrt(n*k) neurons (in this work m = round(sqrt(n*k)) = 268).

Sequences of cytochrome b of 36 organisms have been used to teach the neural network (see point A.1 in Appendix): https://github.com/biopgms/bioattr/blob/main/teaching_sequences.xml. These 36 organisms have been selected from a wide spectrum of evolution, from primitive organisms to evolutionarily advanced organisms. Selected in this way 36 cytochrome b sequences can be considered as pattern sequences necessary to recognize the other, examined organisms. The supervised learning technique with the on-line backpropagation algorithm has been used for teaching the neural network. This technique allows the neural network to be taught very effectively^30^. A teaching output for the first sequence (i.e. the cytochrome b sequence of Bacteria {#1}) was equal to "0000…0001". For the other sequences used for teaching, "1" was shifted to the left, i.e. a teaching output for the second sequence (i.e. for the cytochrome b sequence of Green alga {#2}) was equal to "0000…0010". A teaching output for the 36th sequence (i.e. the cytochrome b sequence of Four-horned antelope {#36}) was equal to "1000…0000". In this way, the obtained results at the each of 36 outputs are in the range [0, 1] and inform about recognized similarities of examined organism to 36 organisms used to teach ANN (where value 0 means the minimum recognized similarity and value 1 means the maximum recognized similarity). ANN had been teaching (for learning rate = 0.3 and momentum = 0.1) until Root Mean Squared Error (RMSE) was less than 0.001. Teaching process time to decrease the RMSE less than 0.001 was approximately 6 days on computer IBM HS23 (2CPU Xenon E5–2650 × 4 Core, 2.00 GHz, RAM = 12 GB, HDD = 50 GB). The teaching process has been carried out in parallel 50 times, each time for different random generated five-positional binary numbers that code each character in the sequences that have been used to teach the neural network. In this way, 50 versions of the neural network have been obtained (that can be downloaded from: http://staff.uz.zgora.pl/akaspers/bioattr/saved_NNs.zip), each version of neural network taught using a different amino-acid coding. Then, each examined organism had been recognized 50 times by each version of the neural network. The final results of recognition have been calculated as an arithmetic mean of the results obtained in the given outputs of the neural networks.

### Semihomologous approach

The semihomologous method assumes that the one-point mutation in the codons of compared amino-acids is the most frequent mechanism occurring in homologous proteins. This method posits close relations between amino-acids and their codons for the analysis of various relationships between proteins. The semihomologous approach allows for improving (compared to standard homologous approach) the accuracy of protein sequence comparison which avoids result misinterpretations^23–27^. The semihomologous algorithm assumes the existence of the following position types when comparing two amino-acids: (a) homology positions (marked as "R")—positions with comparison of the same amino-acids; (b) transition positions (marked as "#") – semihomologous positions with transition type one-point mutations in the codons of the compared amino-acids; (c) transversion positions (marked as "$")—semihomologous positions with transversion type one-point mutations in the codons of compared amino-acids.

## Results and discussion

In this section the results of two approaches are presented to examine evolution of selected organisms. These two approaches use artificial neural network (ANN) and semihomologous Dot-Matrix methods, that are implemented as the EvolutionXXI and dotPicker programs.

### Human evolution—ANN approach

**Figure 1:**
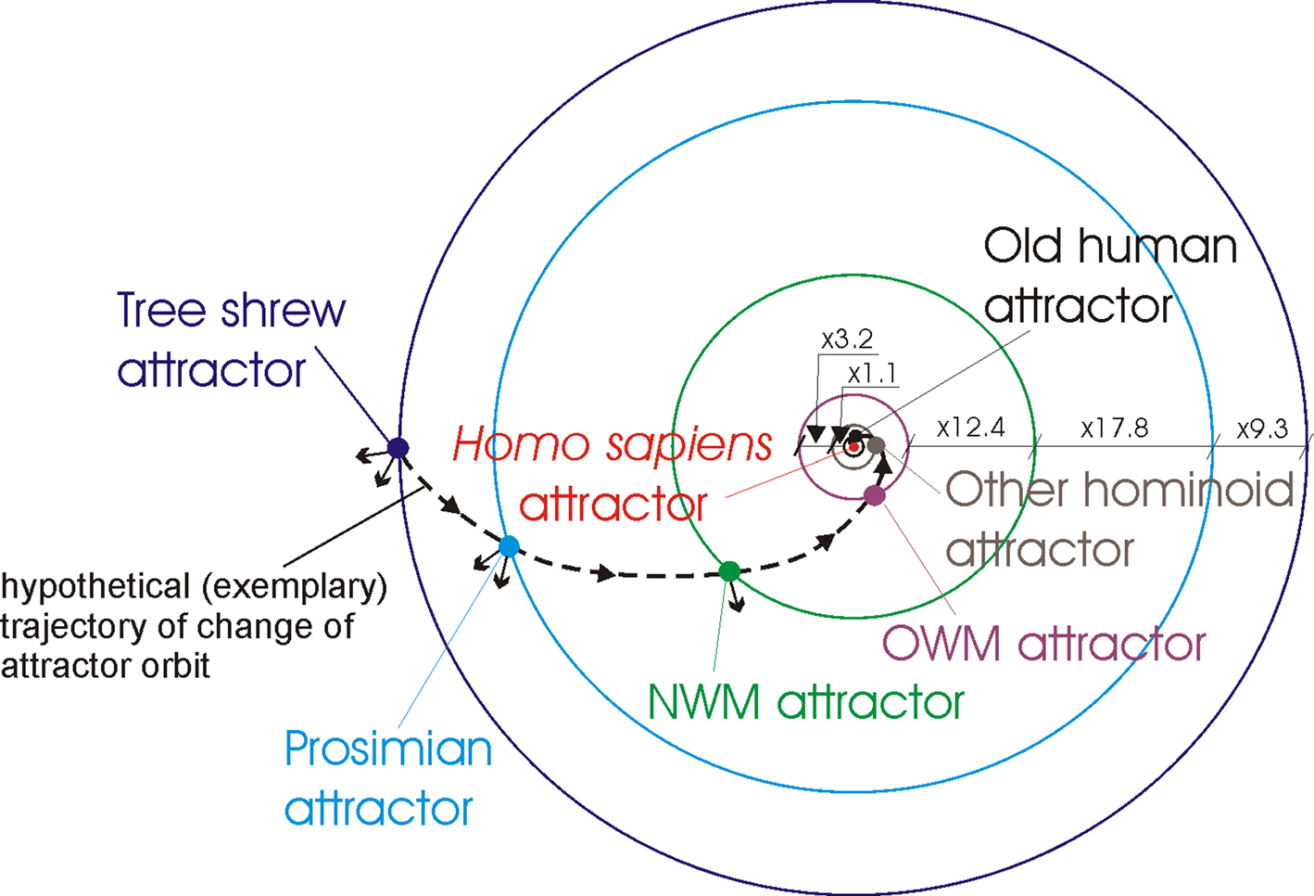
Orbits and genome attractors during human evolution. Tree shrew attractor orbit is disturbed most strongly by the attraction in the Asian rat {#26} (with average similarity to all organisms in the attractor equals to 0.0025) and Domestic sheep {#35} (0.0014) directions—schematically presented by small arrows from the attractor. Prosimian attractor orbit is disturbed most strongly by the attraction in the Horse {#32} (0.0037) and Chinese hare {#24} (0.0013) directions. NWM attractor orbit is disturbed most strongly by the attraction in the Domestic sheep {#35} (0.0015) direction.

The human evolution has been examined taking into account evolution of monkeys (i.e. Tree shrews, Prosimians, New World Monkeys (NWM), Old World Monkeys (OWM)), Other hominoids (here: hominoids except for Old humans) and Old humans (here: *Homo heidelbergensis*, *Homo sapiens ssp. Denisova*, *Homo sapiens neanderthalensis*). The obtained evolutionary similarities (recognized using ANN) between selected organisms from these groups and *Homo sapiens* are presented in Tables 1–6 (see Appendix). Analysis of the results points out the evolutionary distances between these groups, i.e. it appears that the organisms of these groups are trapped in the local genome attractors. Assuming that these attractors are in the orbits (additionally see Remark 1), the distance between the orbits of Old human attractor and *Homo sapiens* attractor is very small with a distance factor equal to 1.0013 (i.e. *Homo sapiens* attractor orbit/Old human attractor orbit ≈ 1.0013). The distance between the orbits of Other hominoid attractor and Old human attractor is bigger with a distance factor equal to 1.1. The distances between the other orbits are much bigger with distance factors equal accordingly to: 3.2, 12.4, 17.8, 9.3 (Fig. 1). The small arrows pointing from the genome attractors to the outside of the orbits schematically represent disturbances (i.e. attractions to the other organisms to which similarities have been recognized by ANN) of the attractor orbits (Fig. 1).

#### Remark 1

The distances between orbits represent evolutionary distances between genome attractors without taking into account intermediate/invisible stages of evolution (note: distances between orbits are calculated and presented as distance factors, i.e. inner orbit/outer orbit, see Tables 1–6 and Tables 7–10 in Appendix). Taking into account the impact of the intermediate/invisible stages of evolution requires applying distance corrections that increase distance factors (see “Introduction”). The location of the genome attractors in the orbits allows for this correction to be presented in a very flexible way, i.e. by appropriately shifting (left/right) the genome attractors along the orbits, it is possible to present the increase or decrease of this correction.

### Human evolution—semihomologous approach

**Figure 2:**
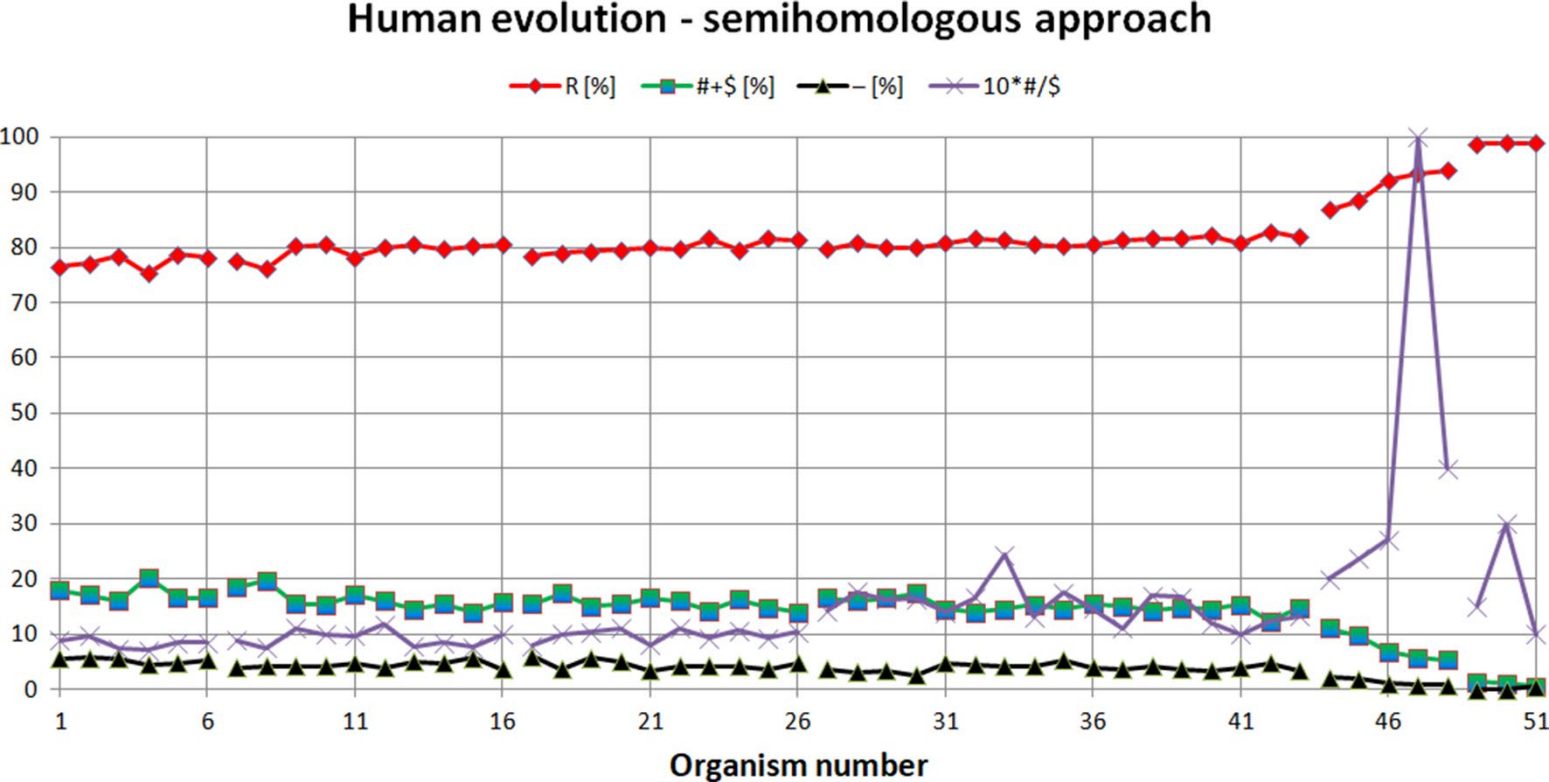
Human evolution examined using semihomologous approach. The organisms presented on the X-axis are set in the order determined by the neural network from the organism most evolutionarily distant from *Homo sapiens* (organism number 1 on the X-axis, i.e. *Tupaia belangeri*), to the organism closest to human (organism number 51 on the X-axis, i.e. *Homo heidelbergensis*). "R"—homologous positions, "# + $"—semihomologous positions (sum of positions with transitions and transversions), "−"—positions with two or three point mutations, "#/$" factor—ratio # to $.

The semihomologous approach has been used to examine Human evolution. The results are presented in Fig. 2 and in Tables 1–6 (see Appendix). Note: the organism numbers shown on the X-axis correspond to the numbers presented in the column 'Organism no. in Fig. 2′ in Tables 1–6 (see Appendix).

**Figure 3:**
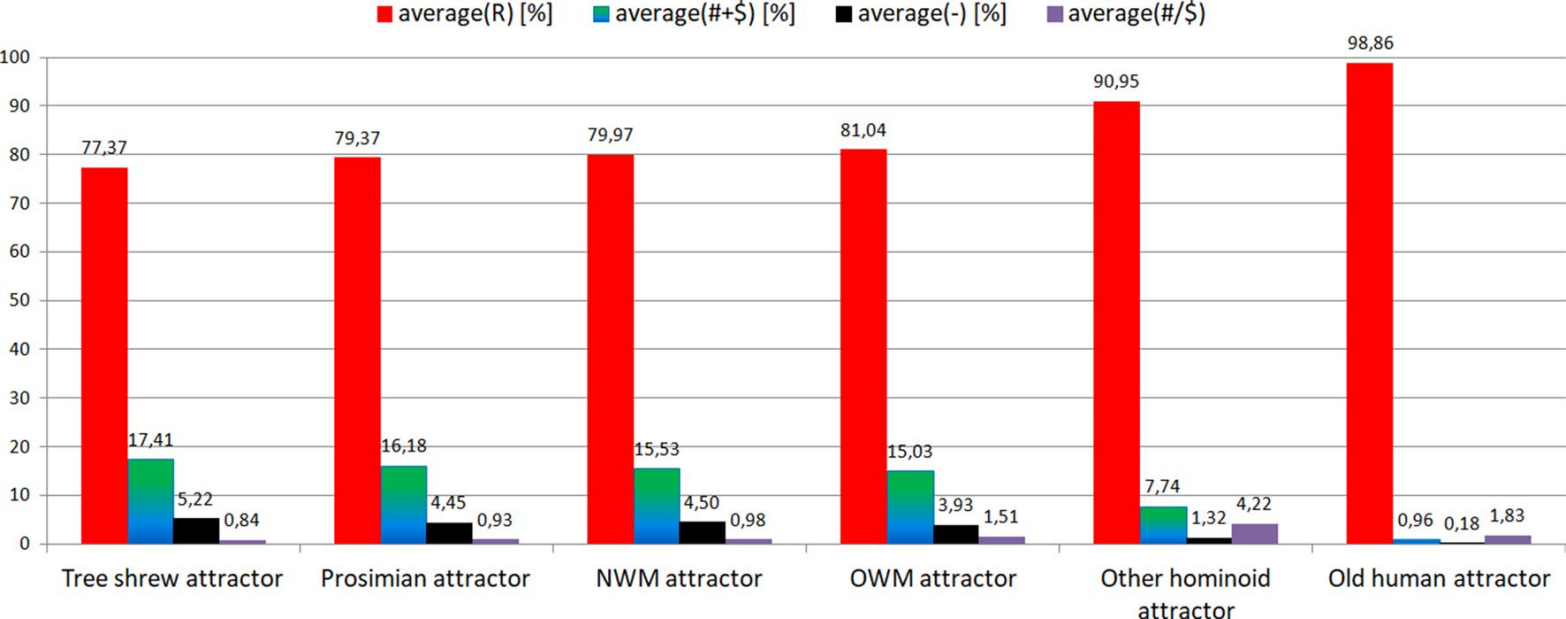
Characteristics of the genome attractors during Human evolution using semihomologous approach.

From Fig. 2 it is noticeable that the semihomologous approach used to examine Human evolution confirms the results obtained using ANN. During Tree shrew, Prosimian, NWM and OWM development, the numbers of positions (comparing to *Homo sapiens*) with two point and three point mutations (i.e. "−" positions) and semihomologous positions (i.e. "# + $" positions) very slowly decreases. The number of homologous positions (i.e. "R" positions) slowly increases. Other hominoid development is characterized by faster decreasing the numbers of "−" and "# + $" positions and faster increasing "R" positions. Old humans attractor is characterized by very small number of "−" and "# + $" positions and very high number of "R" positions. Semihomologous characteristics: for Tree shrew attractor (average("R") = 77.37[%]; average("# + R") = 17.41[%];

average("-") = 5.22[%]; average("#/$") = 0.84); for Prosimian attractor (79.37[%]; 16.18[%]; 4.45[%]; 0.93); for NWM attractor (79.97[%]; 15.53[%]; 4.5[%]; 0.98); for OWM attractor (81.04[%]; 15.03[%]; 3.93[%]; 1.51); for Other hominoid attractor (90.95[%]; 7.74[%]; 1.32[%]; 4.22); for Old human attractor (98.86[%]; 0.96[%]; 0.18[%]; 1.83). It should be noted that the average ("#/$") factor increases during evolution of these organisms and is less than 1 for attractors of Tree shrews, Prosimians, NWM and is bigger than 1 for attractors of OWM, Other hominoids, Old humans (Fig. 3).

It should also be noted that using only the semihomologous approach it is impossible to separate most groups of organisms (i.e. Tree shrews, Prosimians, NWM, OWM) because the number of homologous and semihomologous positions and positions with two and three point mutations is almost the same for these groups (see Remark 2).

#### Remark 2

One of the key properties of neural networks is a generalization allowing for the correct recognition and classification of previously unseen/unknown patterns (i.e. sequences that have not been used for teaching when referring to the considerations presented in this article). The advantage of using ANN (comparing to semihomologous approach) for separation of organisms into groups that represent attractors may be due to the fact that during recognition ANN takes into account not only amino-acid similarities/dissimilarities but also the distribution of these similarities/dissimilarities, i.e. the recognition using ANN may resemble recognizing images formed ("painted") by sequences.

### Yeast evolution – ANN approach

**Figure 4:**
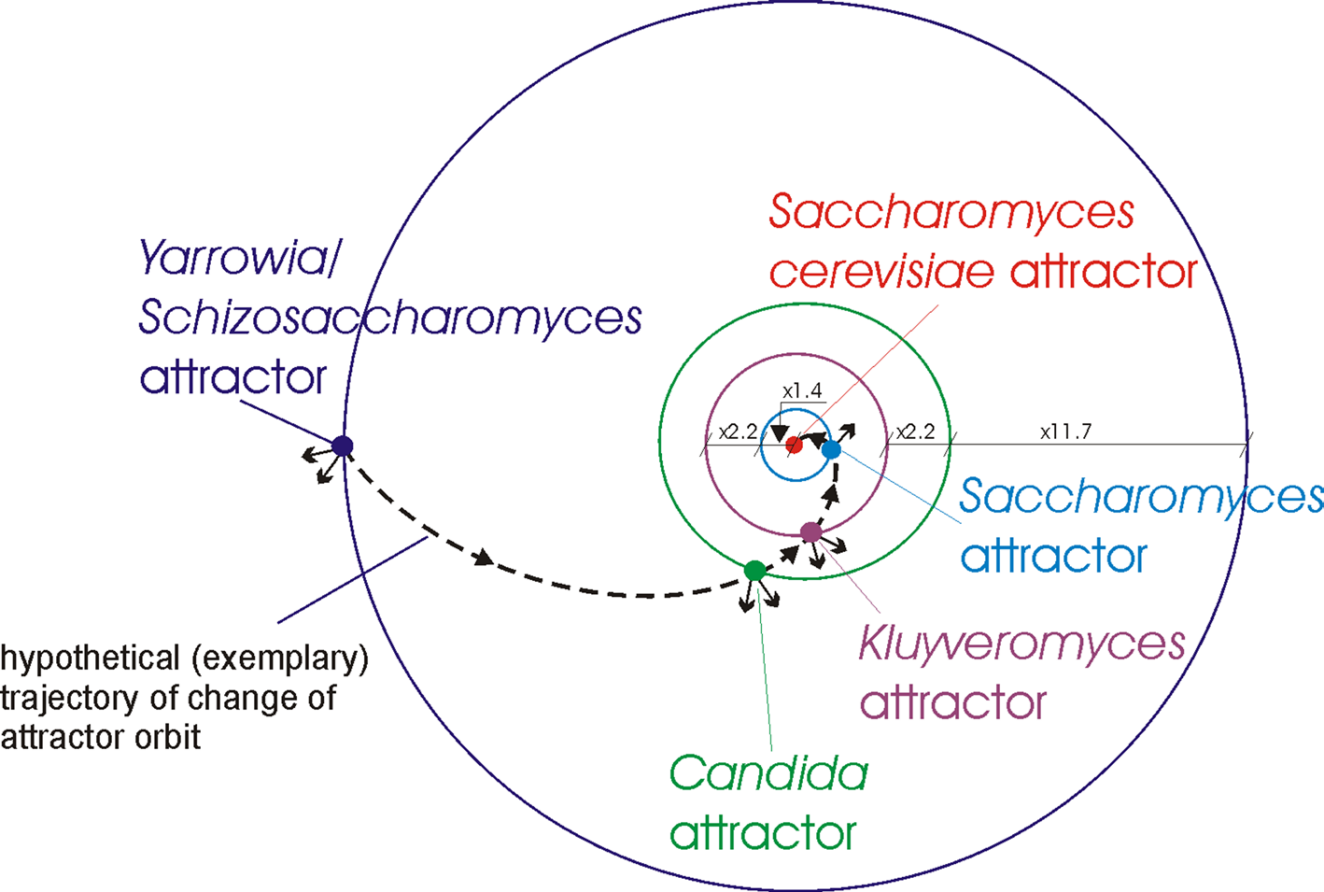
Orbits and genome attractors during yeast evolution. *Yarrowia*/*Schizosaccharomyces* attractor orbit is disturbed most strongly by the attraction in the Poplar mushroom {#8} (with average similarity to all organisms in the attractor equals to 0.006) and Green alga {#2} (0.004) directions—schematically presented by small arrows from the attractor. *Candida* attractor orbit is disturbed most strongly by the attraction in the Poplar mushroom {#8} (0.008) and Green alga {#2} (0.002) directions. *Kluyveromyces* attractor orbit is disturbed most strongly by the attraction in the Poplar mushroom {#8} (0.004) and Green alga {#2} (0.002) directions. *Saccharomyces* attractor orbit is disturbed by the attraction in the Poplar mushroom {#8} (0.001) direction.

The yeast evolution has been examined taking into account evolution of several strains of yeasts. The obtained evolutionary similarities (recognized using ANN) between *Saccharomyces cerevisiae* and selected other yeasts are presented in Tables 7–10 (see Appendix). The yeasts during evolution are trapped in four genome attractors, i.e. *Saccharomyces*, *Kluyveromyces*, *Candida*, *Yarrowia*/*Schizosaccharomyces* attractors (Tables 7–10 in Appendix). Analysis of the results points out the evolutionary distances between these groups, i.e. it appears (similar as in the case of human evolution) that the organisms of these groups are trapped in the local genome attractors. Assuming that these attractors are in the orbits (additionally see Remark 1), the distance between the orbits of *Saccharomyces* attractor and *Saccharomyces cerevisiae* attractor can be considered as average with a distance factor equal to 1.4 (i.e. *Saccharomyces cerevisiae* attractor orbit/*Saccharomyces* attractor orbit ≈ 1.4). The distances between the other orbits are bigger with distance factors equal accordingly to: 2.2, 2.2, 11.7 (Fig. 4).

### Yeast evolution – semihomologous approach

**Figure 5:**
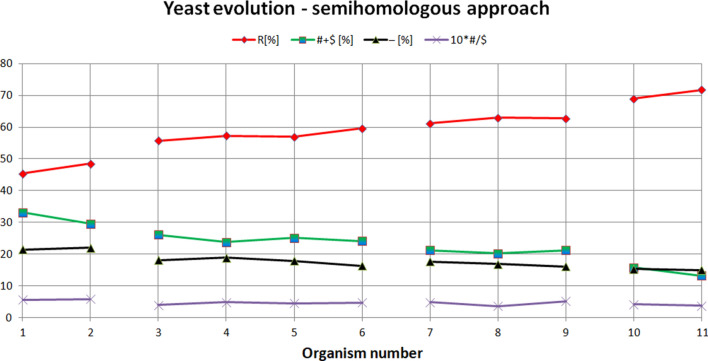
Yeast evolution examined using semihomologous approach. The organisms presented on the X-axis are set in the order determined by the neural network from the organism most evolutionarily distant from *Saccharomyces cerevisiae* (organism number 1 on the X-axis, i.e. *Schizosaccharomyces pombe*), to the organism closest to *Saccharomyces cerevisiae* (organism number 11 on the X-axis, i.e. *Saccharomyces paradoxus*). "R"—homologous positions, "# + $"—semihomologous positions (sum of positions with transitions and transversions), "−"—positions with two or three point mutations, "#/$" factor—ratio # to $.

The semihomologous approach has been used to examine yeast evolution. The results are presented in Fig. 5 and in Tables 7–10 (see Appendix). Note: the organism numbers shown on the X-axis correspond to the numbers presented in the column 'Organism no. in Fig. 5’ in Tables 7, 8, 9, 10 (see Appendix).

**Figure 6:**
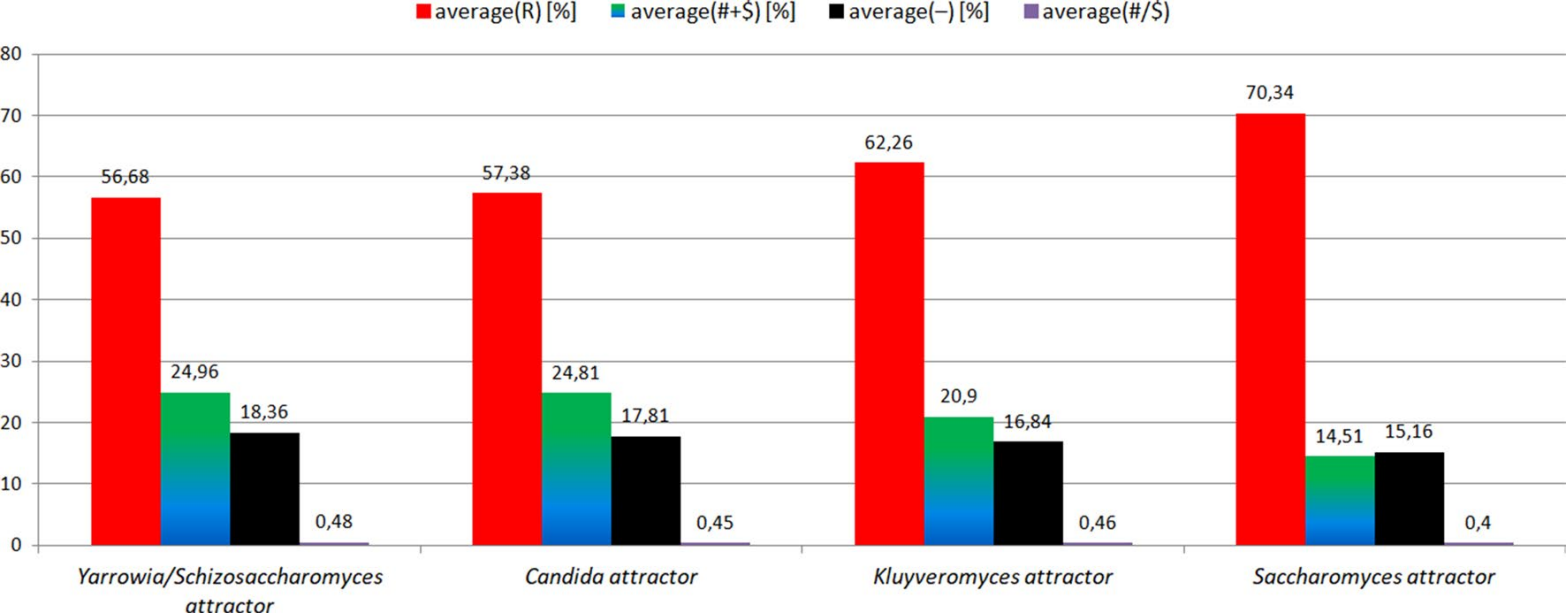
Characteristics of the genome attractors during yeast evolution using semihomologous approach.

From Fig. 5 it is noticeable that the semihomologous approach used to examine yeast evolution confirms the results obtained using ANN. During yeast evolution the numbers of positions (comparing to *Saccharomyces cerevisiae*) with two point and three point mutations (i.e. "−" positions) and semihomologous positions (i.e. "# + $" positions) decreases. The number of homologous positions (i.e. "R" positions) increases. Semihomologous characteristics: for *Yarrowia/Schizosaccharomyces* attractor (average("R") = 56.68[%]; average("# + R") = 24.96[%]; average("−") = 18.36[%]; average("#/$") = 0.48); for *Candida* attractor (57.38[%]; 24.81[%]; 17.81[%]; 0.45); for *Kluyveromyces* attractor (62.26[%]; 20.9[%]; 16.84[%]; 0.46); for *Saccharomyces* attractor (70.34[%]; 14.51[%]; 15.16[%]; 0.4) (Fig. 6). It should be noted that in this case ANN enables a much clearer separation of organisms into individual groups compared to semihomologous approach (see Remark 2).

### Study on evolution of the other, selected organisms

The ANN approach has not detected clear direction in the evolution of bats, hippopotamuses, sirenians, rhinoceroses, squirrels (https://github.com/biopgms/bioattr/blob/main/other_organisms.pdf). This may indicate that these organisms are trapped in local genome attractors (bearing in mind that organisms from a wide range of evolution have been used to teach the neural network).

The recognition of evolution using ANN points out that orbit of Bat attractor (checked for exemplary bats: *Aeorestes cinereus*, *Anoura caudifer*, *Aselliscus stoliczkanus*, *Brachyphylla cavernarum*, *Casinycteris argynnis*, *Cynopterus brachyotis*, *Epomophorus gambianus*, *Epomops dobsonii*, *Eptesicus serotinus*, *Glischropus aquilus*, *Hipposideros lylei*, *Myotis altarium*, *Plecotus macrobullaris*, *Ptenochirus jagori*, *Rhinolophus ferrumequinum tragatus*, *Scotonycteris bergmansi*, *Sphaerias blanfordi*, *Vampyressa pusilla*) is disturbed most strongly by the attraction in the direction of Horse {#32} (with average similarity to all organisms in the attractor equals to 0.002), Gray wolf {#30} (0.002) and Chinese hare {#24} (0.002).

Orbit of Hippopotamus attractor (checked for exemplary hippopotamus: *Hexaprotodon liberiensis*, *Hippopotamus amphibius*) is disturbed most strongly by the attraction in the direction of Blue whale {#33} (0.006), Brown bear {#29} (0.004) and Four-horned antelope {#36} (0.002).

Orbit of Sirenia attractor (checked for exemplary mantas: *Dugong dugon*, *Hydrodamalis gigas*, *Trichechus manatus*) is disturbed most strongly by the attraction in the direction of African bush elephant {#31} (0.005) and African lion {#28} (0.004).

Orbit of Rhinoceros attractor (checked for exemplary rhinoceros: *Rhinoceros sondaicus*, *Rhinoceros unicornis*) is disturbed most strongly by the attraction in the direction of African bush elephant {#31} (0.005) and African lion {#28} (0.004).

Orbit of Squirrel attractor (checked for exemplary squirrel: *Sciurus aestuans*, *Sciurus lis*, *Sciurus niger*, *Sciurus vulgaris*) is disturbed most strongly by the attraction in the direction of Chinese hare {#24} (0.003).

### Attractors during development of transformed cells

**Figure 7:**
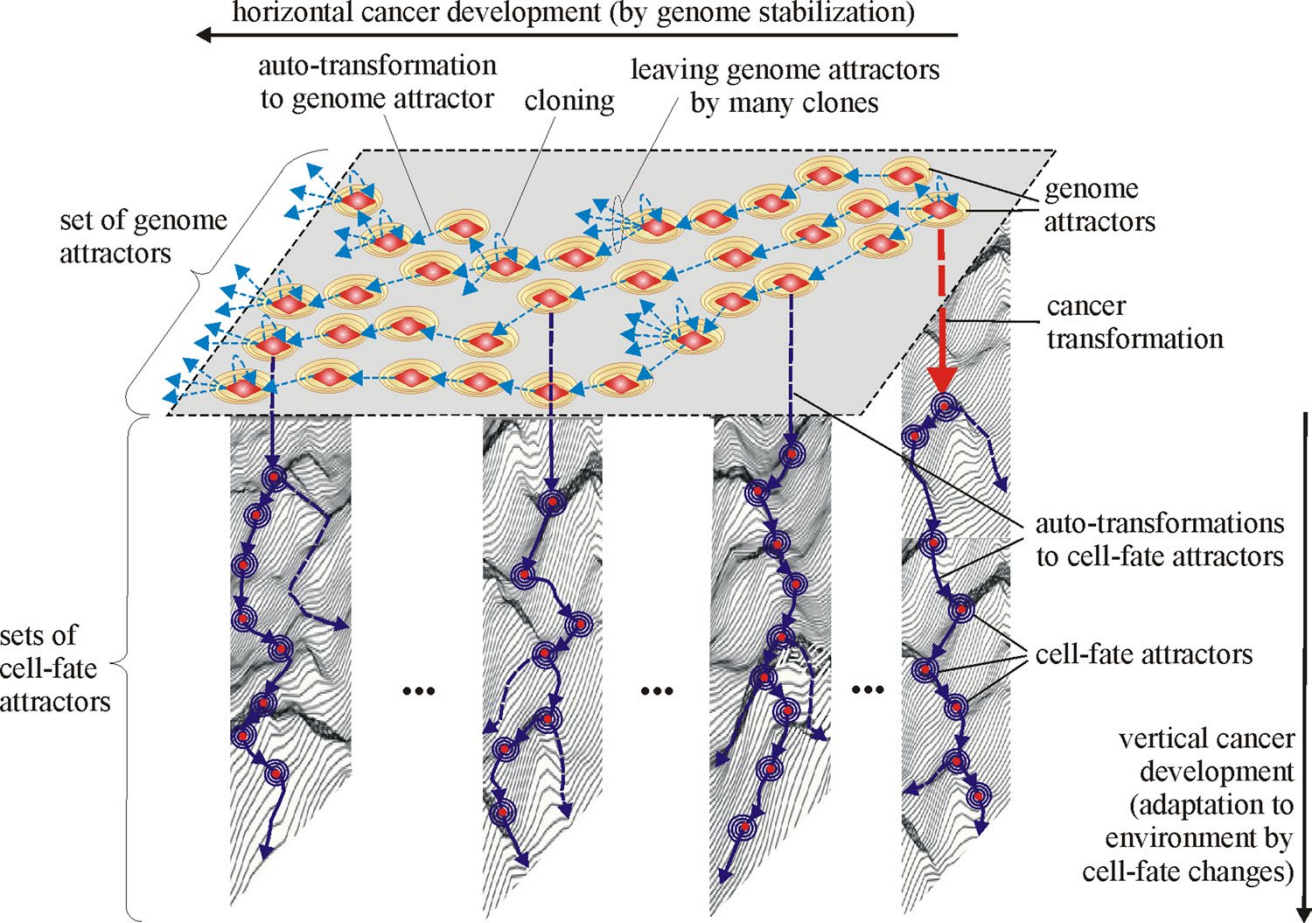
Horizontal and vertical cancer development in view of the new universal model of cancer transformation and cancer development. Cancer transformation activates, among others, cloning which causes that many clones can leave (because of occurrence of genome chaos) current genome attractor (GA) and attain new/different genome attractors by auto-transformation to genome attractor. The clones are kept alive after attaining new genome attractors by establishing appropriate new cell-fates. After attaining a new genome attractor, random mutations caused by elevated ROS level can lead to: (**a**) genome chaos occurrence and a change of genome attractor (macro-cellular evolution) or (**b**) destabilization of current cell-fate and a change of cell-fate attractor (micro-cellular evolution). Note: micro-cellular evolution can also occur without mutations, only as a result of cell-fate adaptation to changes in environment (vertical cancer development). It should be also noted that the figure shows only example sets of cell-fate attractors (as well as the presentation of the cloning process and leaving genome attractors by many clones are also limited to only a few cases) to ensure the clarity of this figure. In accordance with schemas of cancer development (Figs. 10, 11), vertical development occurs, among others, after each change of genome attractor.

Cells after cancer transformation display a series of paradoxes—in this view, there is a need for a more system-based framework to understand the complex phenomena associated with the development of cancer^32^. This section presents a new (based on the attractor concept) system approach to the universal explanation of cancer transformation and cancer development. It is known, that mitochondria play especially important role during the development of cancer that, in certain cancer settings, can act as neoplastic drivers by generating high levels of oncometabolites that are able to change the genomic and epigenomic landscape of the cell^33^. Taking into account that the cancer genome can be considered as a complex network of mutually regulating genes, this network can lose stability and can also, under certain conditions, produce hundreds of stable equilibrium states termed as attractors^34–36^. In this view, the new universal model of cancer transformation and development has been established and schematically presented in Fig. 7. This new model can be considered a significant extension, improvement and unification of the proposal presented in^37^. There can be distinguished two types of attractors (i.e. cancer cell-fate attractors and genome attractors) in accordance with this new model (Fig. 7).

**Figure 8:**
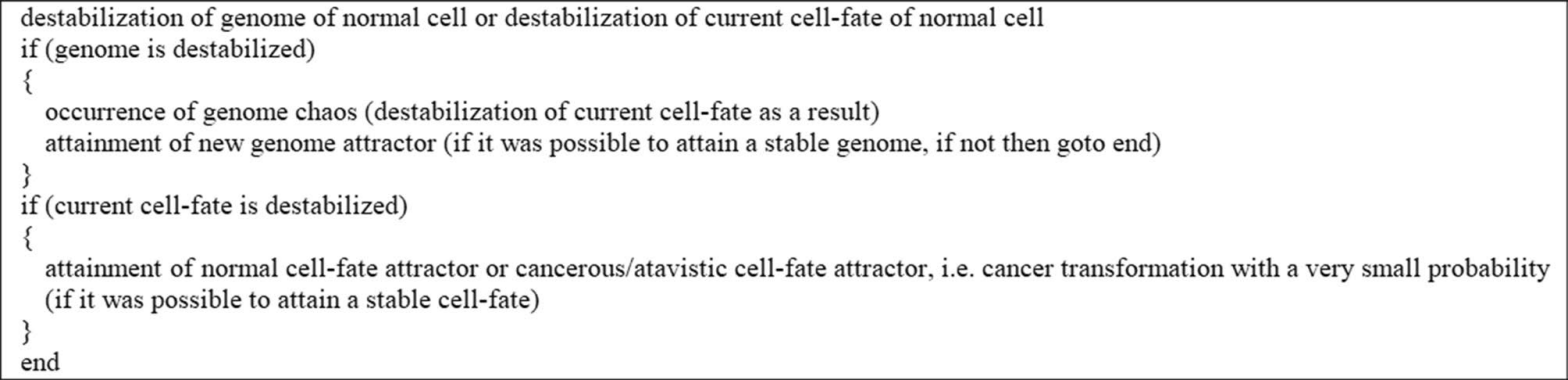
Schema of cancer transformation.

**Figure 9:**
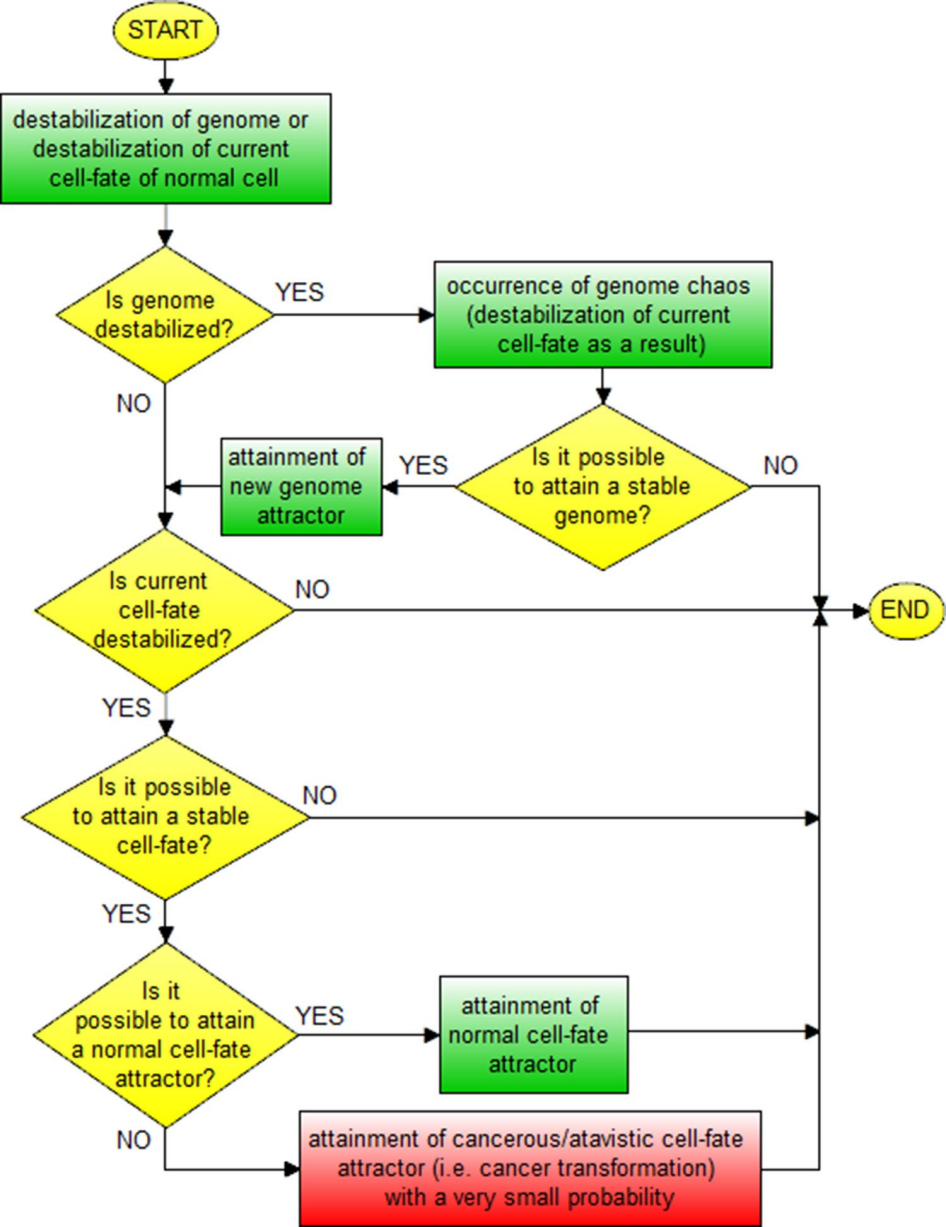
Block schema of cancer transformation.

**Figure 10:**
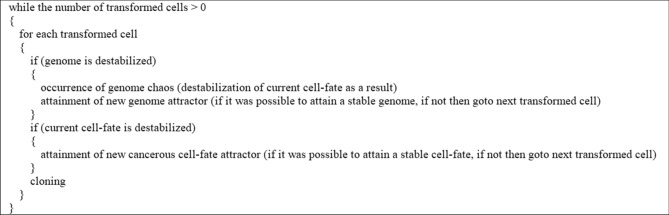
Schema of cancer development.

**Figure 11:**
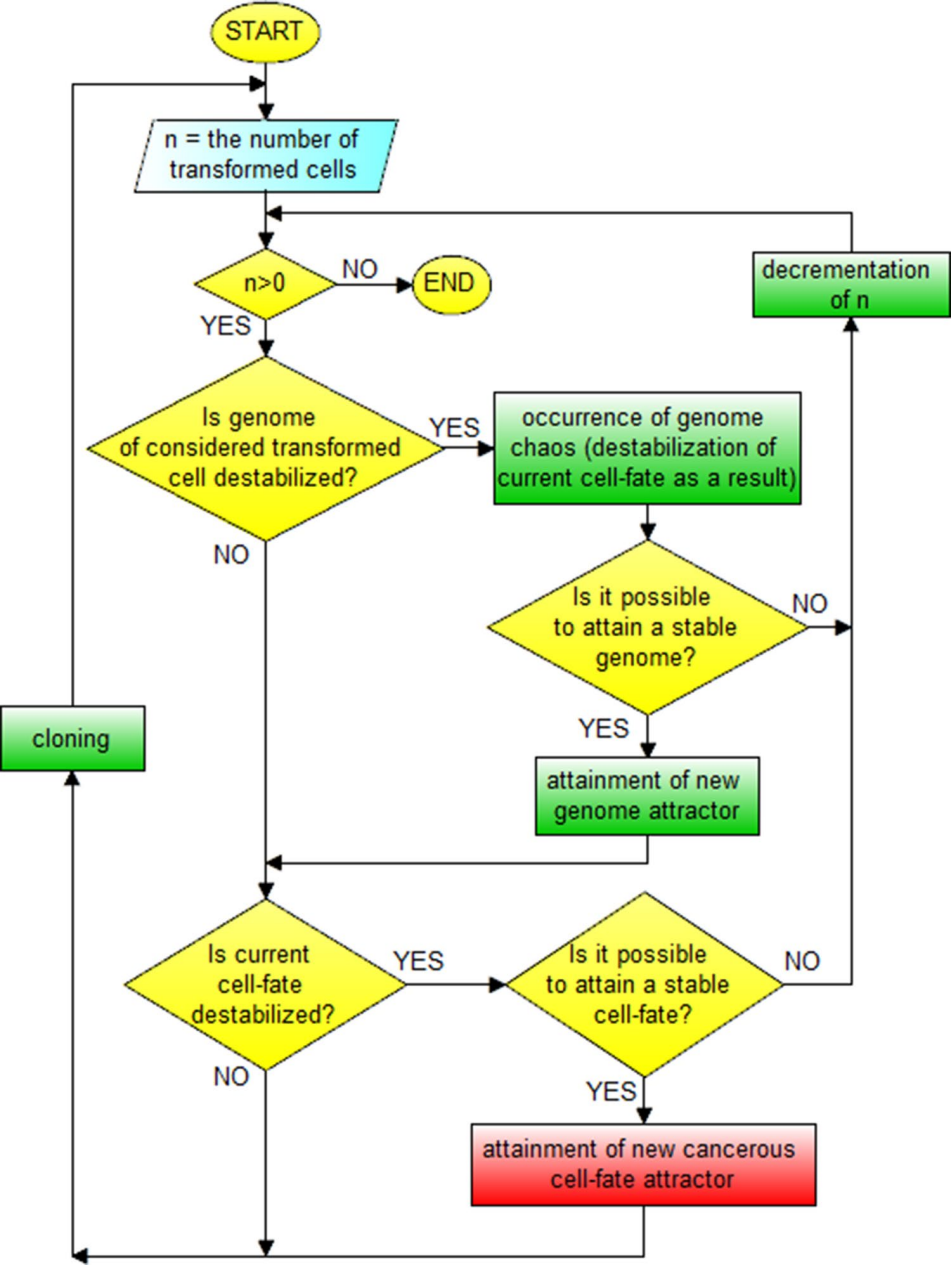
Block schema of cancer development.

In accordance with Fig. 7 two types of cancer development can be distinguished during cancer development, i.e. the vertical and horizontal development of cancer. Vertical cancer development is based on step-by-step changes of cell-fate attractors. Horizontal cancer development is based on step-by-step changes of genome attractors. The vertical development always occurs during cancer development, while the horizontal development of cancer is optional, i.e. it may or may not occur (additionally see schemas of cancer transformation and development presented in Figs. 8, 9, 10, 11). This indicates that cancer development can occur as a clearly vertical development (during which vertical development occurs only). Cancer development can also occur as a mix of vertical development and horizontal development—during that type of development, horizontal development is always followed by vertical development (see Figs. 10, 11).

### Cancer cell-fate attractors

Cancer development, presented in Fig. 7 as vertical (i.e. from top to down) development, occurs through step-by-step changes of cell-fates by cancer clones. This type of cancer development occurs without a change of genome attractor and can occur without DNA mutations (see Remark 3).

#### Remark 3

Vertical development of cancer is driven by 'non-genetic instability', i.e. it is driven by instability of the phenotype^38^. Vertical cancer development occurs as an adaptation of cell-fates to external (environment) factors. It can also occur as an adaptation of cell-fates to internal factors, among others, random changes of genome (but without occurrence of genome re-organization) by elevated level of ROS. That means that vertical cancer development occurs without a change of genome attractor and can occur without DNA mutations (additionally see schemas of cancer development (Figs. 10, 11)).

After destabilization of current cell-fate (i.e. destabilization of current gene expression program), the processes of establishing new cell-fate (i.e. establishing new gene expression program) and its stabilization are activated^39^. The process of cell-fate stabilization is schematically presented in Fig. 7 as auto-transformation to cell-fate attractor (i.e. establishing stable cell-fate means that cell-fate attains cell-fate attractor). From this point of view, cell-fate attractor can be considered as a bioenergetic state toward which a running gene expression program (coded in DNA) strives to attain stability. In view of unified cell bioenergetics (UCB), overenergization of mitochondria is one of the reasons for cancer transformation and then cancer development (for details see^37,40,41^). Overenergization can cause the switch of current cell-fate to cancerous/atavistic cell-fate^37^. The aim of the activation of cancerous/atavistic cell-fate is to prevent overenergized mitochondria against an excessive amount of ROS (additionally see Remark 4). The reversal of cancer cells towards early protists was suggested previously^42–46^ and formulated by some authors as the atavistic theory of cancer^47–53^. Switching to cancerous cell-fate (i.e. cancer transformation) causes, among others, activation of atavistic gene expression program and, as a result, cell disruption from the body, occurrence of the Warburg effect and emergence of the other cancer hallmarks that include: sustained proliferative signaling, evasion of growth suppressors, resistance to cell death, enablement of replicative immortality, energy metabolism list-reprogramming, evasion of immune destruction, inducement of angiogenesis, and the activation of invasion and metastasis^54^. Cancer development leads also to the outgrowth of a clonally derived population of cancer cells. Moreover, tumors contain a repertoire of recruited cells that contribute to the acquisition of the aforementioned hallmark traits by creating a 'tumor microenvironment'^54^.

#### Remark 4

In accordance with unified cell bioenergetics (UCB), a stimulation of aerobic fermentation can inhibit mitochondrial NADH (mtNADH) increase and as a result inhibit ROS production. From this point of view, the Warburg effect represents a cellular defense strategy that reduces the oxidative stress status of the cells^55^. According to current observations, the Warburg effect occurs even in the presence of completely functioning mitochondria and the glycolytic contribution to total ATP production does not generally exceed 50–60%^56,57^. For this reason OXPHOS (i.e. oxidative phosphorylation) substantially contributes to ATP production after cancer transformation^58^. This phenomenon allows maintenance of charging mitochondria with NADH in many cancer cell types. Moreover, gaining energy through highly intensive aerobic glycolysis that occurs after cancer transformation can additionally inhibit overenergized mitochondria discharge. As a result ROS levels are increased in many types of cancer cells, that is consistent with other research findings^59^.

As an example of cancer development through changes of cell-fate attractors can be given development which occurs after stimulation (being trapped in genome attractor) of the MCF-7 breast-cancer cell line by HRG (heregulin)^39^. It is known that HRG-stimulation induces cell differentiation^60^. HRG activates the ErbB receptor with sustained extracellular signal regulated kinase (ERK) activity. As a result, after stimulation of MCF-7 breast-cancer cells by HRG, the destabilization of current cell-fate occurs, what induces the cells leave current cell-fate attractor to be trapped in another cell-fate attractor with stabilization of new cell-fate as an outcome^39^. This type of cancer development can be considered as an adaptation (by cell-fate changes) to changes in the environment. From Fig. 7 it is visible that a lot of different, stable phenotypes can be obtained for each genotype. This hallmark of the way cancer develops can be described as one-genotype-many-phenotypes, with a paradigm of 1:n mapping. The ability of the regulatory control structures of a system to produce more than one stable system state is called multi-stability^38^.

### Cancer genome attractors

Cancer development, presented in Fig. 7 as horizontal (i.e. from right to left) development, is based on step-by-step changes of genome attractors by cancer clones. This type of cancer development is optional (it may or may not occur). Occurrence of this type of development can be considered as a supporting (important) component of the development of some cancers leading to, among others, polyploidy and aneuploidy. Horizontal cancer development is associated with genome re-organization and is driven by genome instability (GIN). In accordance with unified cell bioenergetics (UCB), cancerous mitochondria generate an excessive amount of reactive oxygen species (ROS) (for details see^37,40,41^). Thus, cancer cells exhibit increased levels of ROS compared to normal cells. High level of ROS causes an increase of the number of random DNA mutations^61^. As a result, the probability of changes (by these mutations) of DNA fragments that code the mechanisms responsible for monitoring the integrity of the genome increases^61^. A defect in the regulation of these mechanisms can result in GIN. GIN includes chromosome instability (CIN), chromosome structure instability (CSI), microsatellite instability (MSI) and small structure variations^62,63^. GIN and tumor promoting inflammation are among the hallmarks of cancer^54,64–67^. Aneuploidy (i.e. abnormal numbers of chromosomes that arise through CIN by the persistent loss and gain of whole chromosomes) and chromosome large-scale structural rearrangements (induced by CSI as a consequence of improper repair of DNA damage) are other important features of cancer cells^62^. Both CIN and CSI are associated with advanced stages of cancer development (characterized by, among others, increased resistance to chemotherapy and invasiveness)^62^. The chromosomal changes induced by CIN and CSI provide the driving mechanism that allows cancer cells to sample the genomic landscape^62^. The aim of the sampling is to find an aneuploid karyotype that may be transformative or best suited for growth in stressful environments^62^. This sampling is supported by the cloning mechanism. It should be noted that cancer are clonal for aneuploidy above a threshold^68^. Aneuploidy is a ubiquitous feature of cancer^69^. Generally, aneuploidy can be described as numerical or structural, depending on whether whole chromosomes or portions of chromosomes are gained or lost. Both of these are distinct from polyploidy, in which cells contain more than two complete sets of chromosomes, but always contain an exact multiple of the haploid number, so the chromosomes remain balanced^70^. Aneuploidy and polyploidy occur frequently in tumors^70^. Polyploid cells are known to display greater capacity for adaptation to environmental challenge comparing to their diploid counterparts^47^. GIN leads to occurrence of genome chaos^71^. Genome chaos is a process of complex, rapid genome re-organization that results in the formation of unstable genomes, which is followed by the potential to establish stable genomes^72^. The process of genome stabilization is schematically presented in Fig. 7 as auto-transformation to genome attractor. Establishing a stable genome means that the cell has been trapped in genome attractor. From this point of view, the cancer genome attractor can be considered as a physical state in which a genome attains stability. Occurrence of genome chaos and then genome stabilization cause the emergence of a re-organized genome, i.e. it can be said that a modified organism of the same type is emerging. This modified organism is then kept alive by establishing new cell-fate (i.e. horizontal cancer development is followed by vertical cancer development) that allows it to stay alive.

In view of presented information, schema and block schema of cancer transformation can be depicted as shown in Figs. 8 and 9.

Schema and block schema of cancer development can be depicted as shown in Figs. 10 and 11.

The schemas presented in Figs. 8, 9, 10, 11 along with Fig. 7 explain known controversies related to the development of cancer ^68^:

(i) many carcinogens do not mutate genes;

(ii) there is no functional proof that mutant genes cause cancer;

(iii) mutation is fast but carcinogenesis is exceedingly slow.

In accordance with the presented schemas, cancer transformation (Figs. 8, 9) and development (Figs. 10, 11) can occur without mutations, only as a result of subsequent cell-fate destabilizations (issues (i) and (ii)). It should be added that cancer transformation and then development can also occur as a result of random mutations changing DNA fragments which code mechanisms responsible for monitoring the integrity of the genome, leading to GIN and consequently to genome chaos (with genome re-organization) followed by a change of genome attractor (see the “Cancer genome attractors” section). After cancer transformation, cancer development can also occur both as horizontal cancer development (as a result of subsequent genome destabilizations) followed by vertical cancer development (as a result of subsequent cell-fate destabilizations) (Figs. 7, 10, 11). After destabilization of current cell-fate of normal cell as a result of fast occurring mutations, the cells can undergo cancer transformation and as a result attain cancerous/atavistic cell-fate with a very small probability (Figs. 8, 9), for this reason cancer transformation requires a long time (issue (iii)).

## Conclusions

This work presents new approaches to the separation of organisms into groups that represent attractors (i.e. artificial neural network, semihomologous Dot-Matrix method and unified cell bioenergetics concept). The carried out analyzes point out that pattern recognition by neural network allows for very effective and clear organism separation (see Remark 2). Semihomologous Dot-Matrix method confirms the results and is a good method for detailed attractor analyzes. Analysis of the development of normal exemplary organisms (i.e. human and yeasts) points out that the organisms get trapped in the local attractors during evolution (Figs. 1, 4). Because the ROS level in normal cells is moderate, for this reason ROS can stimulate living processes without a big impact on changes of genome attractors. Comparing to attractors of normal cells, cancer attractors are very unstable. In accordance with the proposed new universal model, cancer transformation and then development, can occur without genome re-organization (vertical development in Fig. 7), i.e. step-by-step cancer development, from one cell-fate attractor to the next cell-fate attractor (see Figs. 8, 9, 10, 11 and Remark 3). However, a higher level of ROS in cancer cells (see Remark 4) can also lead to repeated occurrences of genome chaos, and, as a result, permanent changes of genome attractors during cancer development, leading to instability of current gene expressions and (as a result) changes and stabilization of new cell-fates. When viewed from outside, there can be an impression that cancer cells want to escape from the internal ROS flame through permanent changes of genome attractors followed by an adaptation of gene expression to re-organized genome by attaining new cell-fate attractors. In sum, considering this case, cancer transformation and then development can also occur as a result of genome re-organizations (horizontal development in Fig. 7), i.e. step-by-step cancer development, from one genome attractor to the next genome attractor followed by vertical development (see Figs. 8, 9, 10, 11).

## Supplementary Information

Supplementary Information.

## Data Availability

Cytochrome b amino-acid sequences selected for this study were taken from the protein databases NCBI and Protein BLAST. All data generated or analyzed during this study are included in this article.

## Code availability

All calculations presented in this article have been made using the written by the authors EvolutionXXI and dotPicker programs (freely available at https://github.com/biopgms/bioattr). The EvolutionXXI program has been written in Java using the Joone framework. This program contains an implemented neural network. The EvolutionXXI program can be run on any platform with installed Java Virtual Machine (JVM). The dotPicker program has been written in C#. This program contains implemented multidimensional semihomologous Dot-Matrix method. The dotPicker program can be run on Windows with installed .NET Framework.

## References

[1] Harvey PH, Pagel MD (1991). The Comparative Method in Evolutionary Biology.

[2] Damasco A, Giuliani A (2017). A resonance based model of biological evolution. Phys. A.

[3] Lewin R (1993). Complexity: Life at the Edge of Chaos.

[4] Meyer A, Hochachka M (1993). Evolution of mitochondrial DNA in fishes. Biochemistry and Molecular Biology of Fishes.

[5] Rocha-Olivares A, Rosenblatt RH, Vetter RD (1999). Molecular evolution, systematics, and zoogeography of the rockfish subgenus Sebastomus (Sebastes, Scorpaenidae) based on mitochondrial cytochrome b and control region sequences. Mol. Phylogenet. Evol..

[6] Lovejoy NR, de Araújo ML (2000). Molecular systematics, biogeography, and population structure of Neotropical freshwater needlefishes of the genus Potamorrhaphis. Mol. Ecol..

[7] Tsigenopoulos CS, Berrebi P (2000). Molecular phylogeny of North Mediterranean freshwater barbs (genus Barbus: Cyprinidae) inferred from cytochrome b sequences: Biogeographic and systematic implications. Mol. Phylogenet. Evol..

[8] Esposti DM, De Vries S, Crimi M, Ghelli A, Patarnello T, Meyer A (1993). Mitochondrial cytochrome b: Evolution and structure of the protein. Biochim. Biophys. Acta..

[9] Farias IP, Ortı G, Sampaio I, Schneider H, Meyer A (2001). The Cytochrome b gene as a phylogenetic marker: The limits of resolution for analyzing relationships among cichlid fishes. J. Mol. Evol..

[10] Mindell DP, Honeycutt RL (1990). Ribosomal RNA in vertebrates: evolution and phylogenetic applications. Annu. Rev. Ecol. Evol. Syst..

[11] Zardoya R, Meyer A (1996). Evolutionary relationships of the coelacanth, lungfishes, and tetrapods based on the 28S ribosomal RNA gene. Proc. Natl. Acad. Sci. USA.

[12] Van de Peer Y, De Wachter R (1997). Evolutionary relationships among the eukaryotic crown taxa taking into account site-to-site rate variation in 18S rRNA. J. Mol. Evol..

[13] Abouheif E, Zardoya R, Meyer A (1998). Limitations of metazoan 18S rRNA sequence data: Implications for reconstructing a phylogeny of the animal kingdom and inferring the reality of the Cambrian explosion. J. Mol. Evol..

[14] Naylor GJP, Brown WM (1998). Amphioxus mitochondrial DNA, chordate phylogeny, and the limits of inference based on comparisons of sequences. Syst. Biol..

[15] Zardoya R, Cao Y, Hasegawa M, Meyer A (1998). Searching for the closest living relative(s) of tetrapods through evolutionary analyses of mitochondrial and nuclear data. Mol. Biol. Evol..

[16] Tobe SS, Kitchener AC, Linacre AMT (2010). Reconstructing mammalian phylogenies: A detailed comparison of the cytochrome b and cytochrome oxidase subunit i mitochondrial genes. PLoS ONE.

[17] Castresana J (2001). Cytochrome b phylogeny and the taxonomy of great apes andmammals. Mol. Biol. Evol..

[18] Kasperski A, Kasperska R (2016). A new approach to the automatic identification of organism evolution using neural networks. BioSystems.

[19] Hsieh HM (2001). Cytochrome b gene for species identification of the conservation animals. Forensic Sci. Int..

[20] Kumar S, Stecher G, Tamura K (2016). MEGA7: molecular evolutionary genetics analysis version 7.0 for bigger datasets. Mol. Biol. Evol..

[21] Heng HH (2006). Stochastic cancer progression driven by non-clonal chromosome aberrations. J. Cell. Physiol..

[22] Heng HH (2006). Cancer progression by non-clonal chromosome aberrations. J. Cell. Biochem..

[23] Leluk J (2000). A non-statistical approach to protein mutational variability. BioSystems.

[24] Leluk J (2000). Regularities in mutational variability in selected protein families and the Markovian model of amino-acid replacement. J. Comput. Chem..

[25] Leluk J, Konieczny L, Roterman I (2003). Search for structural similarity in proteins. Bioinformatics.

[26] Kasperski A, Kasperska R (2012). A novel method of sequence similarity evaluation in n-dimensional sequence space. Curr. Bioinform..

[27] Kasperski A, Kasperska R (2014). Identification of protein family representatives. Curr. Bioinform..

[28] Ye CJ, Regan S, Liu G, Alemara S, Heng HH (2018). Understanding aneuploidy in cancer through the lens of system inheritance, fuzzy inheritance and emergence of new genome systems. Mol. Cytogenet..

[29] Heng, H. H. *Genome Chaos: Rethinking Genetics, Evolution, and Molecular Medicine*. (Academic Press Elsevier, 2019). ISBN 978-012-8136-35-5 (2019).

[30] Heaton, J. *Introduction to Neural Networks with Java, 1st Edition*, Paperback (2005).

[31] Masters, T. *Practical Neural Network Recipies in C++*. (Academic Press, 1993).

[32] Erenpreisa J, Salmina K, Anatskaya O, Cragg MS (2020). Paradoxes of cancer: survival at the brink. Semin. Cancer Biol..

[33] Cannino G, Ciscato F, Masgras I, Sánchez-Martín C, Rasola A (2018). Metabolic plasticity of tumor cell mitochondria. Front. Oncol..

[34] Kauffman S (1969). Homeostasis and differentiation in random genetic control networks. Nature.

[35] Kauffman SA (1969). Metabolic stability and epigenesis in randomly constructed genetic nets. J. Theor. Biol..

[36] Greaves M, Maley CC (2012). Clonal evolution in cancer. Nature.

[37] Kasperski A, Kasperska R (2018). Bioenergetics of life, disease and death phenomena. Theor. Biosci..

[38] Huang S (2013). Genetic and non-genetic instability in tumor progression: Link between the fitness landscape and the epigenetic landscape of cancer cells. Cancer Metast. Rev..

[39] Zimatore, G., Tsuchiya, M., Hashimoto, M., Kasperski, A. & Giuliani, A. Self-organization of whole gene expression through coordinated chromatin structural transition: Validation of self-organized critical control of genome expression. 10.1101/852681 (2019). 10.1063/5.0058511 PMC10903504 38505632

[40] Kasperski A (2008). Modelling of cells bioenergetics. Acta Biotheor..

[41] Kasperski A, Kasperska R (2013). Selected disease fundamentals based on the unified cell bioenergetics. J. Invest. Biochem..

[42] Erenpreisa J, Kalejs M, Cragg MS (2005). Mitotic catastrophe and endomitosis in tumour cells: An evolutionary key to a molecular solution. Cell Biol. Int..

[43] Erenpreisa J, Wheatley D (2005). Endopolyploidy in development and cancer; "survival of the fattest?". Cell Biol. Int..

[44] Erenpreisa J, Cragg MS, Pontarotti P (2008). Life-cycle features of tumour cells. Evolutionary Biology from Concept to Application.

[45] Erenpreisa J, Cragg MS (2013). Three steps to the immortality of cancer cells: senescence, polyploidy and self-renewal. Cancer Cell Int..

[46] Niculescu VF (2016). Developmental and non developmental polyploidy in xenic and axenic cultured stem cell lines of *Entamoeba invadens* and *E. histolytica*. Insights Stem Cells.

[47] Erenpreisa J (2018). Stress-induced polyploidy shifts somatic cells towards a pro-tumourogenic unicellular gene transcription network. Cancer Hypotheses.

[48] Arguello, F. Atavistic Metamorphosis: A new and logical explanation for the origin and biological nature of cancer: With a discussion on a novel approach to treat cancer. (Samozal, 2011). ISBN-13: 978-1460968994 (2011).

[49] Davies PCW, Lineweaver CH (2011). Cancer tumors as Metazoa 1.0: tapping genes of ancient ancestors. Phys. Biol..

[50] Vincent MD (2011). Cancer: Beyond speciation. Adv. Cancer Res..

[51] Vincent MD (2012). Cancer: A de-repression of a default survival program common to all cells?. BioEssays.

[52] Davies P (2013). Exposing cancer’s deep evolutionary roots. Phys. World.

[53] Lineweaver CH, Davies PCW, Vincent MD (2014). Targeting cancer’s weaknesses (not its strengths): Therapeutic strategies suggested by the atavistic model. BioEssays.

[54] Hanahan D, Weinberg RA (2011). Hallmarks of cancer: The next generation. Cell.

[55] Alfarouk KO (2014). Glycolysis, tumor metabolism, cancer growth and dissemination: A new pH-based etiopathogenic perspective and therapeutic approach to an old cancer question. Oncoscience.

[56] Zu XL, Guppy M (2004). Cancer metabolism: Facts, fantasy, and fiction. Biochem. Biophys. Res. Commun..

[57] Liberti MV, Locasale JW (2016). The Warburg effect: How does it benefit cancer cells?. Trends Biochem. Sci..

[58] Zheng J (2012). Energy metabolism of cancer: Glycolysis versus oxidative phosphorylation (Review). Oncol. Lett..

[59] Zhang BB, Wang DG, Guo FF, Xuan C (2015). Mitochondrial membrane potential and reactive oxygen species in cancer stem cells. Fam. Cancer..

[60] Nagashima T (2007). Quantitative transcriptional control of ErbB receptor signaling undergoes graded to biphasic response for cell differentiation. J. Biol. Chem..

[61] Liou GY, Storz P (2010). Reactive oxygen species in cancer. Free Radic. Res..

[62] Thompson SL, Compton DA (2011). Chromosomes and cancer cells. Chromosome Res..

[63] Yao Y, Dai W (2014). Genomic instability and cancer. J. Carcinog. & Mutagen..

[64] Zetter BR (1998). Angiogenesis and tumor metastasis. Annu. Rev. Med..

[65] Heng HH (2013). Chromosomal instability (CIN): What it is and why it is crucial to cancer evolution. Cancer Metastasis Rev..

[66] Baker SG (2015). A cancer theory kerfuffle can lead to new lines of research. J. Natl. Cancer Inst..

[67] Goodson WH (2015). Assessing the carcinogenic potential of low-dose exposures to chemical mixtures in the environment: The challenge ahead. Carcinogenesis.

[68] Duesberg P, Rasnick D (2000). Aneuploidy, the somatic mutation that makes cancer a species of its own. Cell Motil Cytoskeleton..

[69] Merlo LM, Wang L, Pepper JW, Rabinovitch PS, Maley CC (2010). Polyploidy, aneuploidy and the evolution of cancer. Adv. Exp. Med. Biol..

[70] Zasadil LM, Britigan EMC, Weaver BA (2013). 2n or not 2n: Aneuploidy, polyploidy and chromosomal instability in primary and tumor cells. Semin. Cell Dev. Biol..

[71] Ye CJ, Sharpe Z, Heng HH (2020). Origins and consequences of chromosomal instability: From cellular adaptation to genome chaos-mediated system survival. Genes.

[72] Liu G (2014). Genome chaos: Survival strategy during crisis. Cell Cycle.

