# Supplementary material for: Study on attractors during organism evolution
Source: Sci Rep. 2021 May 5;11:9637. doi: 10.1038/s41598-021-89001-0 (PMC8100110; doi:10.1038/s41598-021-89001-0)
Supplement: Supplementary file 1 — Supplementary Information. [file 41598_2021_89001_MOESM1_ESM.docx]

**Appendix - Study on attractors during organism evolution**

Andrzej Kasperski^1*^, Renata Kasperska^2^

^1^Institute of Biological Sciences, Department of Biotechnology, University of Zielona Gora, Zielona Gora, ul. Szafrana 1, 65-516 Zielona Gora, Poland, e-mail: [A.Kasperski@wnb.uz.zgora.pl](mailto:A.Kasperski@wnb.uz.zgora.pl), ORCID: [0000-0003-2590-6688](https://orcid.org/0000-0003-2590-6688)

^2^Faculty of Mechanical Engineering, University of Zielona Gora, ul. Szafrana 4, 65-516 Zielona Gora, Poland

*To whom correspondence should be addressed.

A.1. Artificial neural network - organisms used to teach.

The cytochrome b amino-acid sequences of the following 36 organisms have been used to teach the artificial neural network (<https://github.com/biopgms/bioattr/blob/main/teaching_sequences.xml>): Bacteria (*Escherichia coli*) {#1}, Green alga (*Chlamydomonas reinhardtii*) {#2}, Pellucid four-tooth moss (*Tetraphis pellucida*) {#3}, Date palm (*Phoenix dactylifera*) {#4}, Maize (*Zea mays*) {#5}, Seep monkey-flower (*Erythranthe guttata*) {#6}, Yeast (*Saccharomyces cerevisiae*) {#7}, Poplar mushroom (*Agrocybe aegerita*) {#8}, Freshwater sponge (*Eunapius subterraneus*) {#9}, Earthworm (*Drawida japonica*) {#10}, Chinese scorpion (*Mesobuthus martensii*) {#11}, Parasitoid wasp (*Diadegma semiclausum*) {#12}, Mosquito (*Anopheles darlingi*) {#13}, Monarch butterfly (*Danaus plexippus*) {#14}, Atlantic awning clam (*Solemya velum*) {#15}, Common octopus (*Octopus vulgaris*) {#16}, Whale shark (*Rhincodon typus*) {#17}, South American lungfish (*Lepidosiren paradoxa*) {#18}, European flounder (*Platichthys flesus*) {#19}, Atif's Lycian salamander (*Lyciasalamandra atifi*) {#20}, Burmese python (*Python bivittatus*) {#21}, Indo-Pacific crocodile (*Crocodylus porosus*) {#22}, Maroon-fronted parrot (*Rhynchopsitta terrisi*) {#23}, Chinese hare (*Lepus sinensis*) {#24}, Golden hamster (*Mesocricetus auratus*) {#25}, Asian rat (*Rattus tanezumi*) {#26}, Human (*Homo sapiens*) {#27}, African lion (*Panthera leo*) {#28}, Brown bear (*Ursus arctos*) {#29}, Gray wolf (*Canis lupus*) {#30}, African bush elephant (*Loxodonta africana*) {#31}, Horse (*Equus caballus*) {#32}, Blue whale (*Balaenoptera musculus*) {#33}, Bactrian camel (*Camelus bactrianus*) {#34}, Domestic sheep (*Ovis aries*) {#35}, Four-horned antelope (*Tetracerus quadricornis*) {#36}.

A.2. Characteristics of attractors of human evolution.

Characteristics of attractors have been determined by recognition (using ANN) and comparison (using semihomologous Dot-Matrix method) of each organism in the considered attractor with *Homo sapiens* (gi|698352801|gb|AIT96887.1). Then, attractor orbits (calculated as average value of the highest and the lowest value recognized by ANN for the organisms in the attractor) and four factors: "R"[%] (calculated as the number of "R" positions presented in percents), "#+$"[%] (i.e. the number of "#+$" positions presented in percents), "-"[%] (i.e. the number of "-" positions presented in percents) and "#/$" have been calculated for each comparison. Characteristics of each attractor have been calculated as average values of the results (i.e. average values of "R"[%], "#+$"[%], "-"[%], and "#/$"). The organisms in the Tables have been presented in the order established using ANN from the highest to the lowest evaluation.

Tab. 1. Characteristics of Old human attractor.

| Organism | ID | ANN evaluation | [R/#/$/-] | R[%] | #+$[%] | - [%] | #/$ | Organism no. in Fig. 2 |
| --- | --- | --- | --- | --- | --- | --- | --- | --- |
| *Homo heidelbergensis* | gi\|568192376\|ref\|YP_008963999.1 | 0.99881 | [376/1/1/2] | 98.95 | 0.53 | 0.53 | 1.00 | 51 |
| *Homo sapiens ssp. Denisova* | gi\|292606421\|ref\|YP_003541057.1 | 0.99857 | [376/3/1/0] | 98.95 | 1.05 | 0.00 | 3.00 | 50 |
| *Homo sapiens neanderthalensis* | gi\|695898490\|ref\|YP_002124314.2 | 0.99855 | [375/3/2/0] | 98.68 | 1.32 | 0.00 | 1.50 | 49 |
| attractor characteristics |  | orbit  0.99868 | [375.67/2.33/1.33/0.67] | 98.86 | 0.96 | 0.18 | 1.83 |  |

Tab. 2. Characteristics of Other hominoid attractor.

| Organism | ID | ANN evaluation | [R/#/$/-] | R[%] | #+$[%] | - [%] | #/$ | Organism no. in Fig. 2 |
| --- | --- | --- | --- | --- | --- | --- | --- | --- |
| *Pan paniscus* | gi\|291464864\|gb\|ADE05938.1 | 0.98902 | [357/16/4/3] | 93.95 | 5.26 | 0.79 | 4.00 | 48 |
| *Pan troglodytes* | gi\|5835134\|ref\|NP_008198.1 | 0.98755 | [355/20/2/3] | 93.42 | 5.79 | 0.79 | 10.00 | 47 |
| *Gorilla grilla grilla* | gi\|195952366\|ref\|YP_002120670.1 | 0.96666 | [350/19/7/4] | 92.11 | 6.84 | 1.05 | 2.71 | 46 |
| *Pongo abelii* | gi\|5835847\|ref\|NP_007847.1 | 0.93853 | [336/26/11/7] | 88.42 | 9.74 | 1.84 | 2.36 | 45 |
| *Symphalangus syndactylus* | gi\|315142495\|gb\|ADT82425.1 | 0.75373 | [330/28/14/8] | 86.84 | 11.05 | 2.11 | 2.00 | 44 |
| attractor characteristics |  | orbit  0.87138 | [345.6/21.8/7.60/5] | 90.95 | 7.74 | 1.32 | 4.22 |  |

Tab. 3. Characteristics of OWM attractor.

| Organism | ID | ANN evaluation | [R/#/$/-] | R[%] | #+$[%] | - [%] | #/$ | Organism no. in Fig. 2 |
| --- | --- | --- | --- | --- | --- | --- | --- | --- |
| *Chlorocebus aethiops* | gi\|66276043\|ref\|YP_238254.1 | 0.39198 | [311/32/24/13] | 81.84 | 14.74 | 3.42 | 1.33 | 43 |
| *Pygathrix nemaeus* | gi\|109689566\|ref\|YP_659511.1 | 0.38965 | [315/26/21/18] | 82.89 | 12.37 | 4.74 | 1.24 | 42 |
| *Macaca mulatta* | gi\|640775062\|gb\|AIA26506.1 | 0.37902 | [307/29/29/15] | 80.79 | 15.26 | 3.95 | 1.00 | 41 |
| *Chlorocebus pygerythrus* | gi\|156471204\|ref\|YP_001427434.1 | 0.35671 | [312/30/25/13] | 82.11 | 14.47 | 3.42 | 1.20 | 40 |
| *Allenopithecus nigroviridis* | gi\|608788081\|ref\|YP_009024930.1 | 0.34060 | [310/35/21/14] | 81.58 | 14.74 | 3.68 | 1.67 | 39 |
| *Colobus angolensis* | gi\|339036155\|gb\|AEJ34078.1 | 0.32831 | [310/34/20/16] | 81.58 | 14.21 | 4.21 | 1.70 | 38 |
| *Chlorocebus sabaeus* | gi\|375298966\|gb\|AFA45398.1 | 0.25990 | [309/30/27/14] | 81.32 | 15.00 | 3.68 | 1.11 | 37 |
| *Erythrocebus patas* | gi\|597955524\|gb\|AHN49837.1 | 0.25425 | [306/35/24/15] | 80.53 | 15.53 | 3.95 | 1.46 | 36 |
| *Miopithecus talapoin* | gi\|375300074\|gb\|AFA46423.1 | 0.25067 | [305/35/20/20] | 80.26 | 14.47 | 5.26 | 1.75 | 35 |
| *Papio hamadryas* | gi\|422711942\|gb\|AFX82186.1 | 0.23320 | [306/33/25/16] | 80.53 | 15.26 | 4.21 | 1.32 | 34 |
| *Colobus guereza* | gi\|75060752\|sp\|Q5BU73.1 | 0.22262 | [309/39/16/16] | 81.32 | 14.47 | 4.21 | 2.44 | 33 |
| *Nasalis larvatus* | gi\|109689552\|ref\|YP_659459.1 | 0.22116 | [310/33/20/17] | 81.58 | 13.95 | 4.47 | 1.65 | 32 |
| *Rhinopithecus roxellana* | gi\|88174287\|gb\|ABD39291.1 | 0.21699 | [307/32/23/18] | 80.79 | 14.47 | 4.74 | 1.39 | 31 |
| *Mandrillus sphinx* | gi\|597955636\|gb\|AHN49941.1 | 0.18541 | [304/41/25/10] | 80.00 | 17.37 | 2.63 | 1.64 | 30 |
| *Mandrillus leucophaeus* | gi\|380254671\|gb\|AFD36272.1 | 0.18216 | [304/39/24/13] | 80.00 | 16.58 | 3.42 | 1.63 | 29 |
| *Cercocebus torquatus* | gi\|608788067\|ref\|YP_009024917.1 | 0.16599 | [307/39/22/12] | 80.79 | 16.05 | 3.16 | 1.77 | 28 |
| *Papio ursinus* | gi\|435856048\|ref\|YP_007316947.1 | 0.14474 | [303/37/26/14] | 79.74 | 16.58 | 3.68 | 1.42 | 27 |
| attractor characteristics |  | orbit  0.26836 | [307.94/34.06/23.06/14.94] | 81.04 | 15.03 | 3.93 | 1.51 |  |

Tab. 4. Characteristics of NWM attractor.

| Organism | ID | ANN evaluation | [R/#/$/-] | R[%] | #+$[%] | - [%] | #/$ | Organism no. in Fig. 2 |
| --- | --- | --- | --- | --- | --- | --- | --- | --- |
| *Aotus azarai* | gi\|529217151\|ref\|YP_008378877.1 | 0.03204 | [309/27/26/18] | 81.32 | 13.95 | 4.74 | 1.04 | 26 |
| *Alouatta seniculus* | gi\|685167412\|gb\|AIN94967.1 | 0.02975 | [310/27/29/14] | 81.58 | 14.74 | 3.68 | 0.93 | 25 |
| *Saguinus midas* | gi\|165907593\|gb\|ABY73335.1 | 0.02851 | [302/32/30/16] | 79.47 | 16.32 | 4.21 | 1.07 | 24 |
| *Aotus trivirgatus* | gi\|305691004\|gb\|ADM64762.1 | 0.02648 | [310/26/28/16] | 81.58 | 14.21 | 4.21 | 0.93 | 23 |
| *Saguinus imperator sub* | gi\|305958531\|gb\|ADM73232.1 | 0.02635 | [303/32/29/16] | 79.74 | 16.05 | 4.21 | 1.10 | 22 |
| *Ateles hybridus* | gi\|685167384\|gb\|AIN94946.1 | 0.02284 | [304/28/35/13] | 80.00 | 16.58 | 3.42 | 0.80 | 21 |
| *Callithrix jacchus* | gi\|75065116\|sp\|Q8M5M3.1 | 0.02028 | [302/31/28/19] | 79.47 | 15.53 | 5.00 | 1.11 | 20 |
| *Callithrix pygmaea* | gi\|529217193\|ref\|YP_008378916.1 | 0.02024 | [301/29/28/22] | 79.21 | 15.00 | 5.79 | 1.04 | 19 |
| *Saimiri boliviensis boliviensis* | gi\|75063626\|sp\|Q711L6.1 | 0.01797 | [300/33/33/14] | 78.95 | 17.37 | 3.68 | 1.00 | 18 |
| *Pithecia monachus* | gi\|260065649\|gb\|ACX30261.1 | 0.01110 | [298/26/33/23] | 78.42 | 15.53 | 6.05 | 0.79 | 17 |
| attractor characteristics |  | orbit  0.02157 | [303.9/29.1/29.9/17.1] | 79.97 | 15.53 | 4.50 | 0.98 |  |

Tab. 5. Characteristics of *Prosimian* attractor.

| Organism | ID | ANN evaluation | [R/#/$/-] | R[%] | #+$[%] | - [%] | #/$ | Organism no. in Fig. 2 |
| --- | --- | --- | --- | --- | --- | --- | --- | --- |
| *Eulemur macaco macaco* | gi\|238866959\|ref\|YP_002929437.1 | 0.00232 | [306/30/30/14] | 80.53 | 15.79 | 3.68 | 1.00 | 16 |
| *Perodicticus potto* | gi\|238866875\|ref\|YP_002929347.1 | 0.00187 | [305/23/30/22] | 80.26 | 13.95 | 5.79 | 0.77 | 15 |
| *Avahi cleesei* | gi\|171769518\|sp\|A1YLR8.1 | 0.00147 | [303/27/32/18] | 79.74 | 15.53 | 4.74 | 0.84 | 14 |
| *Otolemur crassicaudatus* | gi\|238809382\|dbj\|BAH69207.1 | 0.00122 | [306/24/31/19] | 80.53 | 14.47 | 5.00 | 0.77 | 13 |
| *Daubentonia madagascariensis* | gi\|597710938\|gb\|AHN16320.1 | 0.00099 | [304/33/28/15] | 80.00 | 16.05 | 3.95 | 1.18 | 12 |
| *Varecia variegata variegata* | gi\|238866973\|ref\|YP_002929463.1 | 0.00090 | [297/32/33/18] | 78.16 | 17.11 | 4.74 | 0.97 | 11 |
| *Lemur catta* | gi\|5762474\|gb\|AAD51117.1 | 0.00067 | [306/29/29/16] | 80.53 | 15.26 | 4.21 | 1.00 | 10 |
| *Propithecus diadema diadema* | gi\|61212342\|sp\|Q5VJ61.1 | 0.00053 | [305/31/28/16] | 80.26 | 15.53 | 4.21 | 1.11 | 9 |
| *Lepilemur septentrionalis* | gi\|146328623\|sp\|Q20FQ1.1 | 0.00050 | [289/32/43/16] | 76.05 | 19.74 | 4.21 | 0.74 | 8 |
| *Loris tardigradus* | gi\|238908480\|ref\|YP_002929334.1 | 0.00010 | [295/33/37/15] | 77.63 | 18.42 | 3.95 | 0.89 | 7 |
| attractor characteristics |  | orbit  0.00121 | [301.6/29.4/32.1/16.9] | 79.37 | 16.18 | 4.45 | 0.93 |  |

Tab. 6. Characteristics of *Tree shrew* attractor.

| Organism | ID | ANN evaluation | [R/#/$/-] | R[%] | #+$[%] | - [%] | #/$ | Organism no. in Fig. 2 |
| --- | --- | --- | --- | --- | --- | --- | --- | --- |
| *Tupaia longipes* | gi\|34980183\|gb\|AAQ84012.1 | 0.00018 | [297/29/34/20] | 78.16 | 16.58 | 5.26 | 0.85 | 6 |
| *Tupaia glis* | gi\|34980171\|gb\|AAQ84006.1 | 0.00018 | [299/29/34/18] | 78.68 | 16.58 | 4.74 | 0.85 | 5 |
| *Dendrogale melanura* | gi\|34980149\|gb\|AAQ83995.1 | 0.00015 | [286/32/45/17] | 75.26 | 20.26 | 4.47 | 0.71 | 4 |
| *Tupaia salatana* | gi\|34980189\|gb\|AAQ84015.1 | 0.00013 | [298/26/35/21] | 78.42 | 16.05 | 5.53 | 0.74 | 3 |
| *Tupaia chinensis* | gi\|34980155\|gb\|AAQ83998.1 | 0.00011 | [293/32/33/22] | 77.11 | 17.11 | 5.79 | 0.97 | 2 |
| *Tupaia belangeri* | gi\|9997013\|ref\|NP_065227.1 | 0.00008 | [291/32/36/21] | 76.58 | 17.89 | 5.53 | 0.89 | 1 |
| attractor characteristics |  | orbit  0.00013 | [294/30/36.17/19.83] | 77.37 | 17.41 | 5.22 | 0.84 |  |

A.3. Characteristics of attractors of yeast evolution.

Characteristics of yeast attractors have been determined by recognition (using ANN) and comparison (using semihomologous Dot-Matrix method) of each organism in the considered attractor with *Saccharomyces cerevisiae* (gi|4837715|emb|CAA24073.2). The organisms in the Tables have been presented in the order established using ANN from the highest to the lowest evaluation.

Tab. 7. Characteristics of *Saccharomyces* attractor.

| Organism | ID | ANN evaluation | [R/#/$/-] | R[%] | #+$[%] | - [%] | #/$ | Organism no. in Fig. 5 |
| --- | --- | --- | --- | --- | --- | --- | --- | --- |
| *Saccharomyces paradoxus* | gi\|830261269\|gb\|AKL83061.1 | 0.78923 | [277/14/37/58] | 71.76 | 13.21 | 15.03 | 0.38 | 11 |
| *Saccharomyces pastorianus* | gi\|224588083\|ref\|YP_002640603.1 | 0.66033 | [266/18/43/59] | 68.91 | 15.80 | 15.28 | 0.42 | 10 |
| attractor characteristics |  | orbit  0.72478 | [271.5/16/40/58.5] | 70.34 | 14.51 | 15.16 | 0.40 |  |

Tab. 8. Characteristics of *Kluyveromyces* attractor.

| Organism | ID | ANN evaluation | [R/#/$/-] | R[%] | #+$[%] | - [%] | #/$ | Organism no. in Fig. 5 |
| --- | --- | --- | --- | --- | --- | --- | --- | --- |
| *Kluyveromyces polysporus* | gi\|212007810\|ref\|YP_001331016.2 | 0.34846 | [242/28/54/62] | 62.69 | 21.24 | 16.06 | 0.52 | 9 |
| *Kluyveromyces delphensis* | gi\|228015406\|ref\|YP_002836194.1 | 0.30735 | [243/21/57/65] | 62.95 | 20.21 | 16.84 | 0.37 | 8 |
| *Kluyveromyces lactis* | gi\|50812095\|ref\|YP_054497.1 | 0.30288 | [236/27/55/68] | 61.14 | 21.24 | 17.62 | 0.49 | 7 |
| attractor characteristics |  | orbit  0.32567 | [240.33/25.33/55.33/65] | 62.26 | 20.90 | 16.84 | 0.46 |  |

Tab. 9. Characteristics of *Candida* attractor.

| Organism | ID | ANN evaluation | [R/#/$/-] | R[%] | #+$[%] | - [%] | #/$ | Organism no. in Fig. 5 |
| --- | --- | --- | --- | --- | --- | --- | --- | --- |
| *Candida castellii* | gi\|228015416\|ref\|YP_002836203.1 | 0.22248 | [230/30/63/63] | 59.59 | 24.09 | 16.32 | 0.48 | 6 |
| *Candida tropicalis* | gi\|533206647\|ref\|YP_008474988.1 | 0.10345 | [220/30/67/69] | 56.99 | 25.13 | 17.88 | 0.45 | 5 |
| *Candida albicans* | gi\|399158038\|gb\|AFP28794.1 | 0.10310 | [221/30/62/73] | 57.25 | 23.83 | 18.91 | 0.48 | 4 |
| *Candida maltosa* | gi\|288903405\|ref\|YP_003434128.1 | 0.07904 | [215/29/72/70] | 55.70 | 26.17 | 18.13 | 0.40 | 3 |
| attractor characteristics |  | orbit  0.15076 | [221.5/29.75/66/68.75] | 57.38 | 24.81 | 17.81 | 0.45 |  |

Tab. 10. Characteristics of *Yarrowia*/*Schizosaccharomyces* attractor.

| Organism | ID | ANN evaluation | [R/#/$/-] | R[%] | #+$[%] | - [%] | #/$ | Organism no. in Fig. 5 |
| --- | --- | --- | --- | --- | --- | --- | --- | --- |
| *Yarrowia lipolytica* | gi\|309773953\|ref\|NP_075443.2 | 0.01526 | [187/42/72/85] | 48.45 | 29.53 | 22.02 | 0.58 | 2 |
| *Schizosaccharomyces pombe* | gi\|2654247\|emb\|CAA38287.1 | 0.01055 | [175/46/82/83] | 45.34 | 33.16 | 21.50 | 0.56 | 1 |
| attractor characteristics |  | orbit  0.01291 | [181/44/77/84] | 56.68 | 24.96 | 18.36 | 0.48 |  |
